# Sex differences in face looking preferences at birth

**DOI:** 10.1186/s13293-026-00976-9

**Published:** 2026-09-01

**Authors:** Yumnah T. Khan, Alex Tsompanidis, Eden Hymanson, Sehanya Wickramanayake, APEX Consortium, Topun Austin, Carrie Allison, Simon Baron-Cohen

**Affiliations:** 1 Autism Research Centre, Department of Psychiatry, University of Cambridge, Cambridge, CB2 8AH UK; 2 Neonatal Intensive Care Unit, Cambridge University Hospitals NHS Foundation Trust, Cambridge, CB2 0QQ UK; 3 Department of Psychology, University of Cambridge, Cambridge, CB2 3EB UK

**Keywords:** Sex differences, Attention, Gaze-tracking, Infancy, Neurodevelopment, Social cognition

## Abstract

While sex differences in behaviour have attracted considerable scientific interest, their underlying causes are complex and remain poorly understood. One approach to understand whether early biological factors might play a partial role is to study newborns, who have limited postnatal experiences. Previous research on neonatal sex differences is extremely limited, and studies that do exist have yielded mixed results, raised methodological concerns, and are yet to be replicated. To assess these gaps, the present study aimed to reevaluate sex differences in neonatal attention. 130 neonates (67 males, 63 females; mean age = 33 h) were presented with a video of a human face and a non-social object. When analysing percentage looking times, on average, females allocated a greater percentage of time looking at the face relative to males (*d* = 0.40). When analysing absolute looking times, females showed a preference for the face compared to the non-social object, while males showed no preference for either. However, no differences were observed *between* males’ and females’ absolute looking times for the face or object, indicating that sex differences are most prominent in the *relative* allocation of attention. Since sex differences in relative attentional patterns are present from the earliest days of life, one possible explanation for this effect may be due to contributions from prenatal factors (e.g., sex hormones). However, alternative explanations may be that these differences reflect general maturational differences between males and females or arise from low-level visual properties of the stimuli rather than their social versus non-social content.

**Supplementary Information:**

The online version contains supplementary material available at https://doi.org/10.1186/s13293-026-00976-9.

## Highlights

Research on neonatal sex differences remains limited and inconsistent, making it challenging to identify the factors that drive behavioural sex differences.

The present study found that female newborns spent a greater percentage of time looking at a face relative to a non-social object compared to male newborns.

Since these differences are present soon after birth, one possible explanation is that prenatal factors (e.g., sex hormone levels) may contribute to sex differences in behaviour, though alternative explanations remain possible.

The findings may help to understand why several psychiatric and neurodevelopmental conditions, such as autism, show sex differences in prevalence.

**Supplementary Information:**

The online version contains supplementary material available at https://doi.org/10.1186/s13293-026-00976-9.

## Abstract (plain language summary)

Previous research suggests that males and females show psychological and behavioural on-average differences, though there remains debate regarding whether these differences emerge purely due to environmental factors (e.g., gender socialisation) or whether biological factors might also play a partial role. Studying newborn babies may help to address this question, since they have limited exposure to the environment. In this study, 130 newborn babies, who were on average 33 hours old, were shown a video of a human face alongside a video of a non-social object. Female newborns spent a greater proportion of their time looking at the face than male newborns, suggesting that sex differences in attention may be present from the first days of life. One possible explanation is that factors acting before birth, such as prenatal sex hormones, may contribute to these differences. However, other explanations are also possible, including differences in developmental maturity between male and female newborns or differences in the visual characteristics of the videos used.

**Supplementary Information:**

The online version contains supplementary material available at https://doi.org/10.1186/s13293-026-00976-9.

## Background

Sex differences in the brain and behaviour have been a longstanding subject of scientific interest [1]. While the existence of such on-average sex differences is fairly well-established, their underlying causes remain to be fully understood in humans [2]. Indeed, a broad range of factors likely contribute, including various sociocultural (e.g., gender-related socialisation), environmental (e.g., nutrition), and maturational factors (e.g., puberty). Sociocultural factors, for instance, play a particularly prominent role as children become embedded in gender-specific societal norms, peer and parental expectations, and cultural values from an early age [3–7]. In addition to this, certain biological factors acting early in life (e.g., prenatal steroid hormone exposure) are also thought to shape sex differences in behaviour [8].

There is increasing evidence to suggest that prenatal biological factors may play an important role [9, 10]. For instance, on-average structural brain differences are present between males and females at birth [11] and begin to emerge during prenatal development [12]. Moreover, pre- and post-natal sex steroid hormones (e.g., androgens and estrogens) are present in different levels in males and females, and variations in these levels have shown associations with later behaviour and cognition [13–16]. While it is now increasingly accepted that behaviour emerges from an interaction between multiple biological, environmental, and sociocultural factors, there remains uncertainty regarding the relative contributions of each. Addressing this question is particularly challenging in humans, where it is difficult to partition the intertwined effects of the multiple factors that may be at play.

One approach to disentangle these effects is to study newborn infants, who have had minimal postnatal life experience. Due to the inherent practical challenges, research investigating behavioural sex differences in newborn infants remains limited. Amongst the few studies that exist within the area, findings have been mixed. Some prior studies report no on-average neonatal sex differences in eye contact [17], contagious crying, or imitation (Karson et al., 2025). However, other studies observing neonatal behaviour have shown that males on average score higher on reactivity domains (e.g., irritability), while females on average score higher on social responsivity domains (e.g., orientation to faces and voices) [18, 19].

In newborns, Connellan et al. [20] is amongst the few studies assessing sex differences in attention. In this study, 102 newborn infants (mean age = 36 h) were presented with two stimuli sequentially: a real, live human face and a “physical-mechanical” mobile object constructed from scrambled facial features. They reported that, on average, females looked significantly longer at the face compared to the object, while males looked significantly longer at the object compared to the face. Due to their limited postnatal life experience, it was argued that the observed sex differences are likely driven by prenatal biological factors. These results have also been interpreted as early precursors of sex differences in empathising (the drive to identify and respond appropriately to the emotions and thoughts of others) and systemising (the drive to analyse and construct a system) [8]. These traits show well-replicated sex differences across life, with females, on average, showing a greater drive towards empathising and males showing a greater drive towards systemising [21].

To date, there have been no attempts to replicate or further investigate the initial findings from the Connellan et al. [20] study, likely due to the challenges in studying newborn infants within the first days of life. Findings from related studies have subsequently been mixed. A recent meta-analysis summarised 20 experiments that measured attention towards human faces within the first month of life and reported no significant overall sex differences in social perception [22]. However, the *post-hoc* power achieved by the meta-analysis was low. As such, the authors concluded that modest sex differences soon after birth cannot be ruled out and emphasised the need for further large-sample studies. Moreover, there was substantial variability in the stimuli and experimental protocols used across studies, and many of the paradigms included in the meta-analysis did not directly contrast attention towards social vs. non-social stimuli. Instead, these studies primarily assessed variation *within* the social domain by examining infants’ attention to a single face (e.g., mother’s face, a live interactor, or a static photo) or discrimination between different faces (e.g., mother vs. stranger, attractive vs. unattractive, upright vs. inverted, eyes open vs. closed). This factor likely explains the inconstancies observed across studies, as it is possible that sex differences in neonatal attention may only become apparent specifically in response to the social vs. non-social stimulus contrast, as observed in Connellan et al. [20].

Additionally, while the study by Connellan et al. [20] is widely cited, various methodological issues have been raised [22–25]. First, some researchers have criticised the use of a live human face, as subtle changes in appearance or movements across participants could have compromised how controlled the stimulus presentation was [23]. Second, because the face used in Connellan et al. [20] was the primary experimenter, unintentional biases in facial expressions or body positioning could have arisen, though all efforts were made to ensure that the experimenter was blind to the sex of the infant [23, 24]. Third, the use of a sequential looking paradigm (i.e., stimulus presented one after another) has also been challenged as some argue that a preferential looking paradigm more accurately captures infant attentional preferences [23, 24]. Lastly, questions have been raised regarding the replicability of the initial findings, since no attempts to replicate the study have been conducted to date.

In light of these considerations and given the mixed literature around the area, the present study aimed to re-evaluate sex differences in neonatal face versus non-social object looking preferences. The design was based on the Connellan et al. [20] study, with a number of changes and extensions to address methodological criticisms raised. These included, for instance: (a) the modification of the stimuli; (b) the use of digital, dynamic videos to ensure controlled, standardised stimulus presentation and to facilitate future replications; and (c) the use of a preferential looking paradigm in order to more robustly capture neonatal attentional preferences. The updated paradigm was implemented in 130 healthy, term-born neonates within an average of the first 33 h (SD = 16.97) after birth.

## Methods

### Participants and recruitment

Newborns were recruited from postnatal wards at Rosie Hospital, Cambridge, UK. The project was given a favourable ethical opinion by the NHS Research Ethic Committee (reference no. 24/LO/0114). Inclusion criteria included birth at or above 37 weeks gestational age, no known health complications, an APGAR score ≥ 8 at 5 min, and a postnatal age less than 72 h to capture the early neonatal period. An APGAR score is a brief assessment of a newborn’s health conducted immediately after birth that evaluates appearance, pulse, grimace, activity, and respiration. A score between 7 and 10 is generally considered healthy [26] and a threshold of ≥ 8 at 5 min is commonly used in neonatal research [27–29].

Parents of eligible newborns were approached on the postnatal ward and provided with information about the study. Written informed consent was obtained from interested parents. In order to minimise parental biases, the research was framed as a broader investigation into birth and familial factors that influence infant looking preferences. While, for transparency, sex differences were acknowledged as one of the general goals of the project, they were not emphasised as a central aim to avoid biasing the parents. For instance, the Participant Information Sheet described the study as follows: “We will look at how newborns’ looking preferences differ depending on birth factors (e.g., mode of delivery, sex assigned at birth), later development (e.g., language), family history (e.g., family members with a neurodivergent diagnosis), and parental characteristics (e.g., their scores on questionnaires).*”* Parents were then debriefed at the end of the study.

**Fig. 1:**
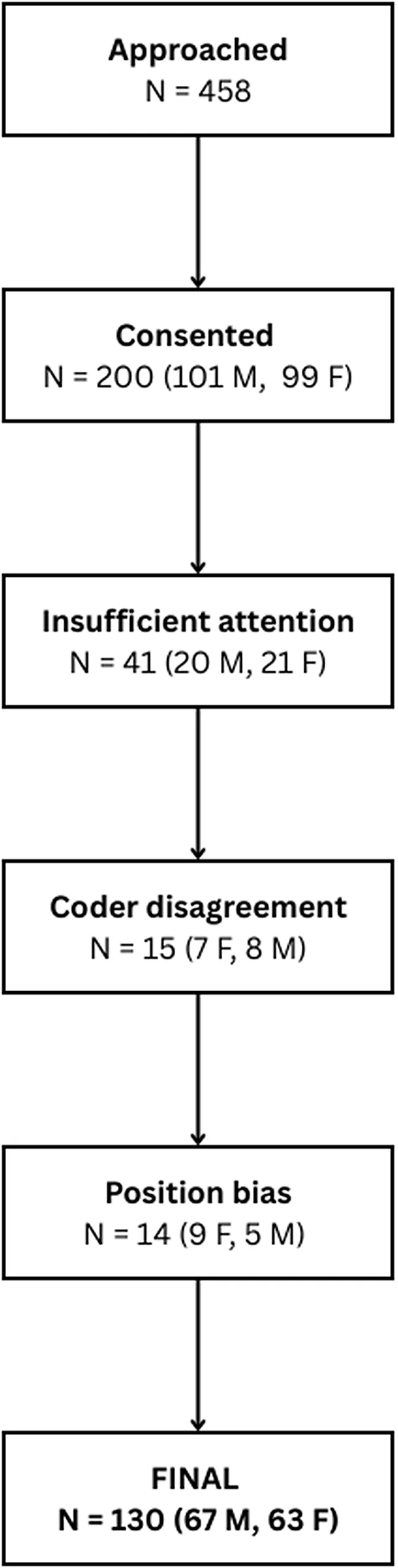
Flow chart summarising sample recruitment and attrition

A total of 200 infants were recruited. Data could not be obtained from 41 infants due to practical constraints, such as the infant not being awake and/or available for testing throughout the day or insufficient visual attention during video presentation (see “Procedure” for criteria). Such attrition rates are not uncommon in neonatal research. Further exclusions were applied to the sample due to discrepancies between the video coders (see video coding procedure and measures) and potential positioning biases. These were defined, based on prior research [30, 31], as the infant looking towards one stimulus > 90% of the time. A detailed breakdown of these exclusions is provided in Fig. 1.

After all exclusions, the final sample consisted of 130 infants (67 males, 63 females) with a mean age of 33 h (SD = 16.97). Additional demographic and birth-related characteristics are presented in Tables 1 and 2. Independent samples *t*-tests and chi-square analyses revealed no significant sex differences across sample characteristics, with the exception of birth weight (males> females), as would be predicted (Supplementary Table 1).

**Table 1:** Sample characteristics (continuous variables)

	Range	Mean (SD) All	Mean (SD) Males	Mean (SD) Females
Postnatal age (hours)	8–72	33.12 (16.51)	32.13 (15.77)	34.17 (17.32)
Gestational age at birth (weeks)	37.0–42.3	39.83 (1.28)	39.92 (1.34)	39.73 (1.22)
Birth weight (grams)	2945–3300	3349.29 (460.56)	3447.61 (473.99)	3244.71 (424.98)
Maternal age (years)	21–43	32.75 (4.30)	33.00 (4.21)	32.48 (4.40)
Paternal age (years)	22–54	34.24 (4.67)	34.38 (3.91)	34.08 (5.40)

Demographic characteristics of the study population for continuous variables, summarised as range and mean (SD) of the total sample and stratified by sex. With the exception of birthweight, no statistically significant differences were identified in any variables

**Table 2:** Sample characteristics (categorical variables)

		All (%)	Males (%)	Females (%)
C-section	Yes	62	34	28
	No	68	33	35
Maternal Education	GCSEs or equivalent	10	4	6
	A-levels or equivalent	15	8	7
	Undergraduate degree	34	17	17
	Postgraduate degree	32	13	19
	Doctorate degree	14	8	6
	Prefer not to say	6	5	1
Paternal education	GCSEs or equivalent	11	7	4
	A-levels or equivalent	19	9	10
	Undergraduate degree	30	12	18
	Postgraduate degree	25	11	14
	Doctorate degree	18	7	11
	Prefer not to say	6	5	1

Demographic characteristics of the study population for categorical variables, summarisedas counts and percentages and stratified by sex. No statistically significant differences wereidentified in any variables

### Stimulus

The stimuli presented in this research aimed to address previous criticisms of the Connellan et al. study [20]. While Connellan et al. [20] presented a live human face and physical object, we chose to use dynamic videos instead. This is because the human face may exhibit subtle changes in appearance or movements across participants [23], so presenting digital stimuli allows for consistent and controlled stimulus presentation. Importantly, remaining blind to the sex of infants is challenging in a hospital setting, and so presenting an actual face could have resulted in unintentional biases in facial expressions or body positioning if the actor had become aware of the infant’s sex. Additionally, we decided to employ a preferential looking paradigm rather than the sequential presentation paradigm used by Connellan et al. [20]. This was in order to address previous critics [23, 24] who argued that preferential looking paradigms are more common [28, 32, 33] and a more effective method to capture infant attentional preferences.

**Fig. 2:**
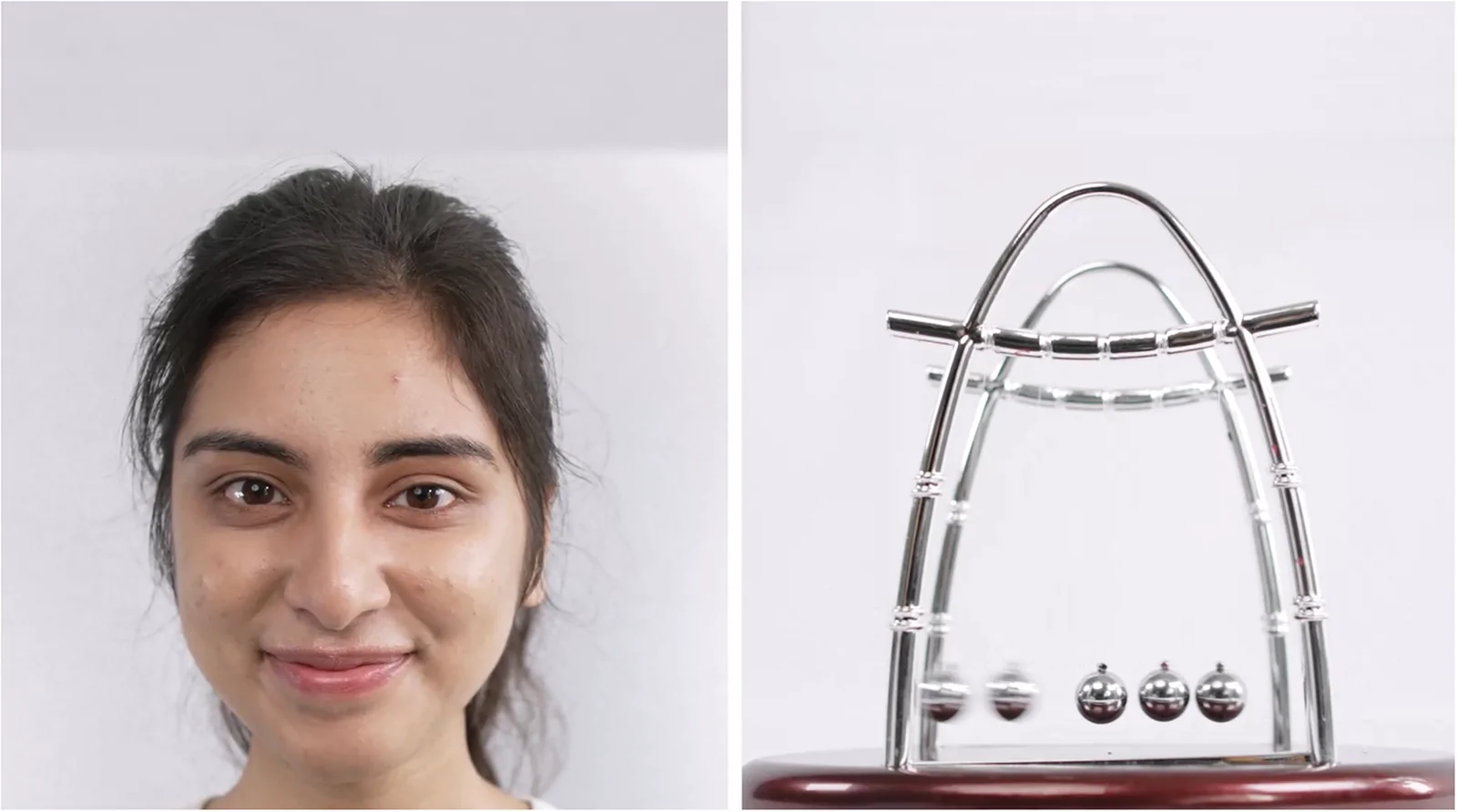
A screen-clipping from the stimulus video depicting the dynamic face and object presented to infants

The social stimulus consisted of a video depicting a human face (female, early 20s, South Asian ethnicity) displaying pleasant facial expressions, as previous studies have indicated that a “still-face” paradigm can be distressing for infants [34, 35]. Given that biological motion is a key indicator of social attention [36], the face exhibited natural facial and head movements, direct eye-contact, and showed periodic smiles to mimic the dynamics of a live interaction. The non-social stimulus comprised a video of moving pendulum balls (Newton’s Cradle) which, unlike the human face, exhibits non-social, systematic, and mechanical motion. One criticism of Connellan et al. [20] was that the scrambled face mobile may not have effectively captured the construct of systemising, which was a background hypothesis behind the research [23]. Systemising is defined as the drive to analyse a system by identifying the rules or patterns governing its behaviour [8]. Although systemising is typically conceptualised as a higher-order ability, an early interest towards systems displaying simple cause-and-effect properties may be present in young infants. The pendulum apparatus was therefore selected as it is a predictable, analysable system, governed by visible cause-and-effect: each ball moves if-and-only-if it is in contact with another ball. Although the pendulum was roughly matched to the size and shape of the face, it was deliberately chosen to be perceptually different to highlight its distinct nature as a non-social stimulus. This was to prevent it from being perceived as a face by a newborn’s neurocognitive system, and to, instead, activate a different non-social processing mechanism. The on-screen width and height of the two stimuli were matched (width: 8.9 cm, height: 13.5 cm). Both stimuli were presented against a white background, separated by an on-screen distance of 7 cm. Both stimuli were brightly lit and filmed under the same lighting conditions. Both stimuli also had high colour contrast to maximise their visual salience for newborns. The full stimulus video can be viewed in the Supplementary Materials and a screen-clipping from the video is presented in Fig. 2 below.

To quantify the overall motion exhibited by the two stimuli, we tracked the positions of the pendulum balls and four facial landmarks (nose, chin, eyes, and mouth) using Vernier Video Analysis (Vernier Science Education, 2023). For the pendulum, we calculated the average velocity across the balls to quantify its overall motion. For the face, we similarly averaged the velocities of the four landmarks to generate a single whole-face motion metric. The pendulum and face exhibited an average on-screen motion of approximately 0.51 cm/s and 0.57 cm/s respectively, indicating that they were broadly matched for motion.

### Procedure

Data were collected between June and December 2024. Researchers closely monitored enrolled infants throughout the day to identify an optimal testing window (i.e., once infants were in a calm, awake, and attentive state, without interfering with the clinical care of the mother or baby). Once ready for testing, the newborns were positioned in the lap of a parent or caregiver 20 cm away from a laptop screen (Lenovo Yoga 6, 13.3”, 1920 × 1080, 16:9) with their gaze aligned to its centre. At this viewing distance, the non-social stimulus subtended ~ 25° of visual angle and the social stimulus ~ 25.7°, with both presented at an eccentricity of ~ 21° from the centre. The video was presented under dim lighting conditions, with the laptop screen set to a moderate level of brightness and it contained no sound. The experimenters and any other people were positioned away from the infant’s line of sight to avoid any distractions. A video camera positioned above the screen (hidden from the view of the infants) recorded the infants’ gaze behaviour to facilitate both real-time monitoring and offline coding.

To initially draw the infant’s attention to the centre of the screen, a red moving dot (with no accompanying sound) was first presented centrally on the screen against a black background. A single trial was initiated as soon as the infant oriented toward this central cue. The video lasted 70 s and simultaneously displayed the two stimuli on opposite halves of the screen, with their left-right positioning randomly counterbalanced across the whole set of participants. In line with Connellan et al. [20], the video was presented in full or for at least 75% of the target duration (52.5 s). If the newborn lost attention or became distressed before this period, the full trial was reinitiated once the infant returned to a calm, attentive state. Additionally, consistent with the approach of infant-control preferential looking paradigms, trials were terminated and reattempted if the infant looked away from the screen for more than 10 consecutive seconds. A trial could therefore proceed as long as all the inattentive breaks were < 10 s and the total period of inattention was < 17.5 s. If this criterion could not be obtained after three attempts, the infant was excluded from the study to minimise burden on the family during this early period.

After the infant’s participation, parents completed a brief questionnaire asking about parental ages, number of siblings, any first-degree relatives diagnosed with neurodevelopmental conditions, and how long ago the infant had their last nap and feed. Families were reimbursed £50 in recognition of the time they devoted to the study during a sensitive period.

### Video coding procedure and measures

As of the time of testing, neither traditional lab-based eye-trackers nor webcam-based eye-trackers were suitable for use in newborn infants. This is because these systems require calibration procedures that are too complex for newborns to follow, and they often struggle with reliable eye detection as newborns have relatively small eyes that are often not fully open. As a result, these methods can result in substantial data loss [37]. Instead, neonatal research has relied on human observers to manually code gaze (Di Giorgio et al., 2016; Kelly et al., 2005; Rigato et al., 2023) by approximating whether an infant is looking towards the left or the right of the screen. Videos were coded offline from the recorded footage. The total duration the infant spent looking at each stimulus (looking time) was computed by summing the durations of all individual looks towards that stimulus. All videos were independently and blindly coded by three trained observers who were not present during the time of testing. The experimenters collecting the data were not blind to the infants’ sex as they worked in close contact with families during recruitment and testing, making blinding logistically unfeasible. To control for this, the independent video coders were blind to the sex of the infant, as well as to which stimulus appeared on which side of the screen (since left-right positioning of the stimuli was counterbalanced across trials).

To achieve reliability, a video was included in the analysis only if at least two coders produced values within ± 10% of one another. Videos failing to meet this inter-coder agreement threshold were excluded from further analysis (see Fig. 1). Additionally, we opted for a consensus coding approach to increase reliability and reduce subjectivity. For each infant, final values for looking time were calculated by averaging the scores from the two coders whose ratings were most closely aligned to one another, identified by having the smallest percentage difference in their coded looking times. Consensus coding is a well-established procedure in developmental and behavioural research, particularly when relying on subjective observational measures. Since there is some degree of subjectivity in using human observers to code eye gaze, there is increased confidence if two coders produce metrics highly consistent with one another. Additionally, selecting two coders that are most consistent with one another minimises the noise introduced by isolated discrepancies from a third coder. Thus, consensus coding maximises the chances of capturing true individual differences in gaze behaviour over measurement noise.

Coders also rated the infant’s overall attentiveness during each trial using a 3-point scale (1 = poor attention, 2 = moderate attention, and 3 = good attention). These ratings were based on the coders’ judgement of how attentive the infants appeared to the video during the trial, taking into account the frequency with which they looked away from the screen and became distracted or fussy. A similar consensus approach was used for the attentiveness ratings, where the final score reflected the majority agreement among coders. Note that all infants in this final sample had already passed the initial inclusion criteria for attentiveness (i.e., gaze diversions from the screen ≤ 10 s and ≥ 75% trial completed). As such, variations in these ratings reflect relative differences within an already attentive sample.

### Statistical analysis

All analyses were pre-planned. To replicate the analysis by Connellan et al. [20], the averaged looking time metric was transformed into a percentage score, calculated by dividing the looking times towards each stimulus by the total looking time across both stimuli. This transformation enables standardised comparisons to account for individual variability in total looking times. An independent samples t-test was then conducted to test for sex differences in percentage looking times. Because the measures are percentages, a difference in face looking time would imply a corresponding difference in object looking time, and therefore only a single test was performed. To determine the presence of a general face preference across the entire sample, a one-sample t-test was conducted to test the percentage looking time towards the face against chance level (0.5).

In addition to percentage looking times, we also analysed absolute looking times via a repeated-measures ANOVA. Sex (male, female) served as the between-subjects variable while stimulus (social or non-social) served as the within-subjects variable. Participant identity was specified as the repeated-measures factor. Alongside these main effects, a sex-by-stimulus interaction was also modelled. When relevant, post-hoc tests were conducted using Tukey’s honestly significant difference test (Tukey’s HSD) controlling for multiple comparisons. Finally, ANCOVAs were conducted on both percentage and absolute looking times to control for postnatal age at testing, gestational age at birth, delivery mode, birth weight, time since last nap and feed, total trial time, number of trials, and time spent looking away from the screen.

## Results

### Inter-rater reliability

First, we assessed inter-rater reliability to evaluate the consistency between coders, which was excellent for the percentage looking times measure (*ICC* = 0.97, *p* < .001). Reliability was good for the absolute looking times measure for faces (*ICC* = 0.86, *p* < .001) and objects (*ICC* = 0.87, *p* < .001).

### Overall attention

The majority of infants (93%) completed the full trial of 70 s. Mean time spent looking away from the screen was 12.87 s (*SD* = 9.42). There were no sex differences in overall trial duration nor in the total time spent looking away from the screen or in the left-right assignment of stimuli (Supplementary Table 1). The majority of infants (105 infants or 80.7%; 57 F, 48 M) required only 1 trial, 15 infants (11%; 4 F, 11 M) required 2 trials, and 10 infants (7.7%; 2 F, 8 M) required 3 trials. There were sex differences in the number of trials conducted, such that females were more likely than males to only require one trial (*χ*^2^(2) = 7.52, *p* = .020). (Supplementary Table 1). In terms of overall attentiveness during the trial as rated by coders, 90 infants (69.2%; 45 F, 45 M) showed good attention, 36 infants (27.7%; 16 F, 20 M) showed moderate attention, and 4 infants (3.1%; 2 F, 2 M) showed poor attention. A chi-square test indicated that there were no sex differences in this attentiveness measure (χ²(2) = 0.32, *p* = .851). Left-right position counterbalancing was roughly similarly distributed between the sexes, with a total of 59 infants (28 F, 31 M) in Condition 1 (right face, left object) and 71 infants (35 F, 37 M) in Condition 2 (left face, right object).

### Percentage looking time

First, we examined whether various birth or testing factors influenced face versus object preferences. Postnatal age at testing, gestational age at birth, delivery mode, birth weight, time since last nap and feed, total trial time, number of trials, and time spent looking away from the screen were not significant predictors of the percentage of time allocated towards the face relative to the object (Supplementary Table 2).

**Fig. 3:**
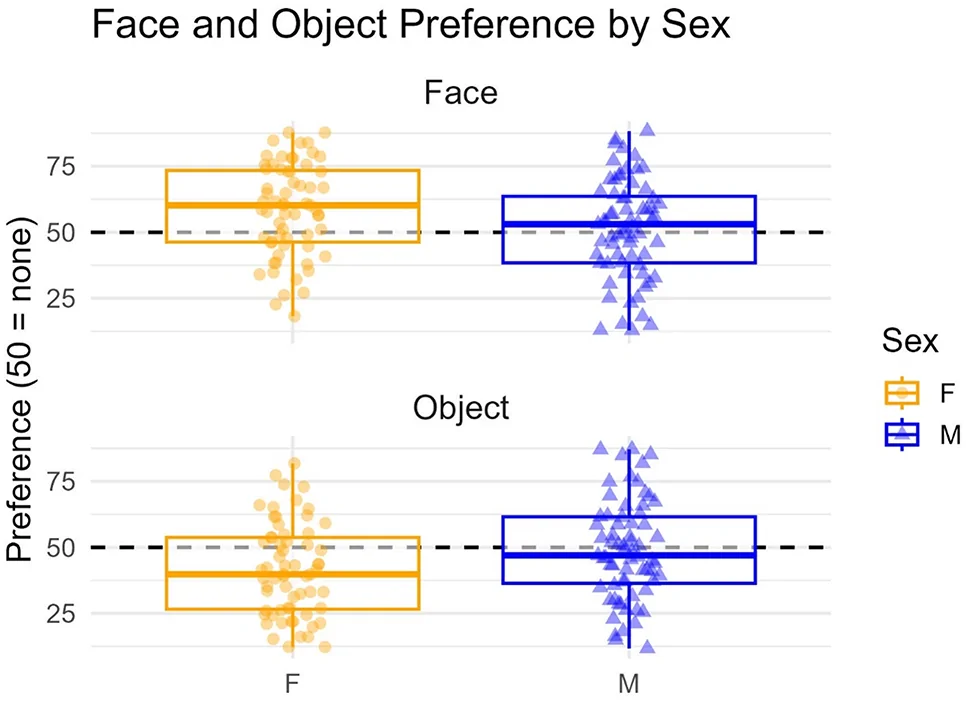
Percentage looking times by sex and stimulus. Boxplots display the distribution of percentage looking times by sex and stimulus. The central horizontal lines on each boxplot indicate the median, the boxes represent the interquartile range (IQR), and the whiskers extend to 1.5 × IQR. The dashed black horizontal line at the centre of the graph marks chance-level (50%). The y-axis is truncated to enhance visualisation

Next, we assessed whether a face preference was present across the entire sample. A one-sample t-test showed that, relative to chance, infants looked at the face for a significantly longer percentage of time than the object (*t*(129) = 33.56, *p* < .001, M = 54.75%, *d* = 0.26). This is in line with the well-established face preference effect. Following the primary analysis of Connellan et al. [20], we then assessed sex differences in the percentage of time allocated towards looking at the face relative to the object. An independent samples t-test showed a significant sex difference (*t*(128) = 2.31, *p* = .023, *d* = 0.40). Relative to the object, females spent a significantly longer percentage of time looking at the face (*M* = 58.53, *SD* = 17.52) compared to males (*M* = 51.20, *SD* = 18.68) (Fig. 3). Since this analysis is on percentages, results of the same test also indicate that males on average looked significantly longer at the object (M = 48.80) compared to females (M = 41.47). The findings remained consistent after controlling for postnatal age at testing, gestational age at birth, delivery mode, birth weight, time since last nap and feed, total trial time, number of trials, and time spent looking away from the screen (Supplementary Table 3).

### Absolute looking times

**Fig. 4:**
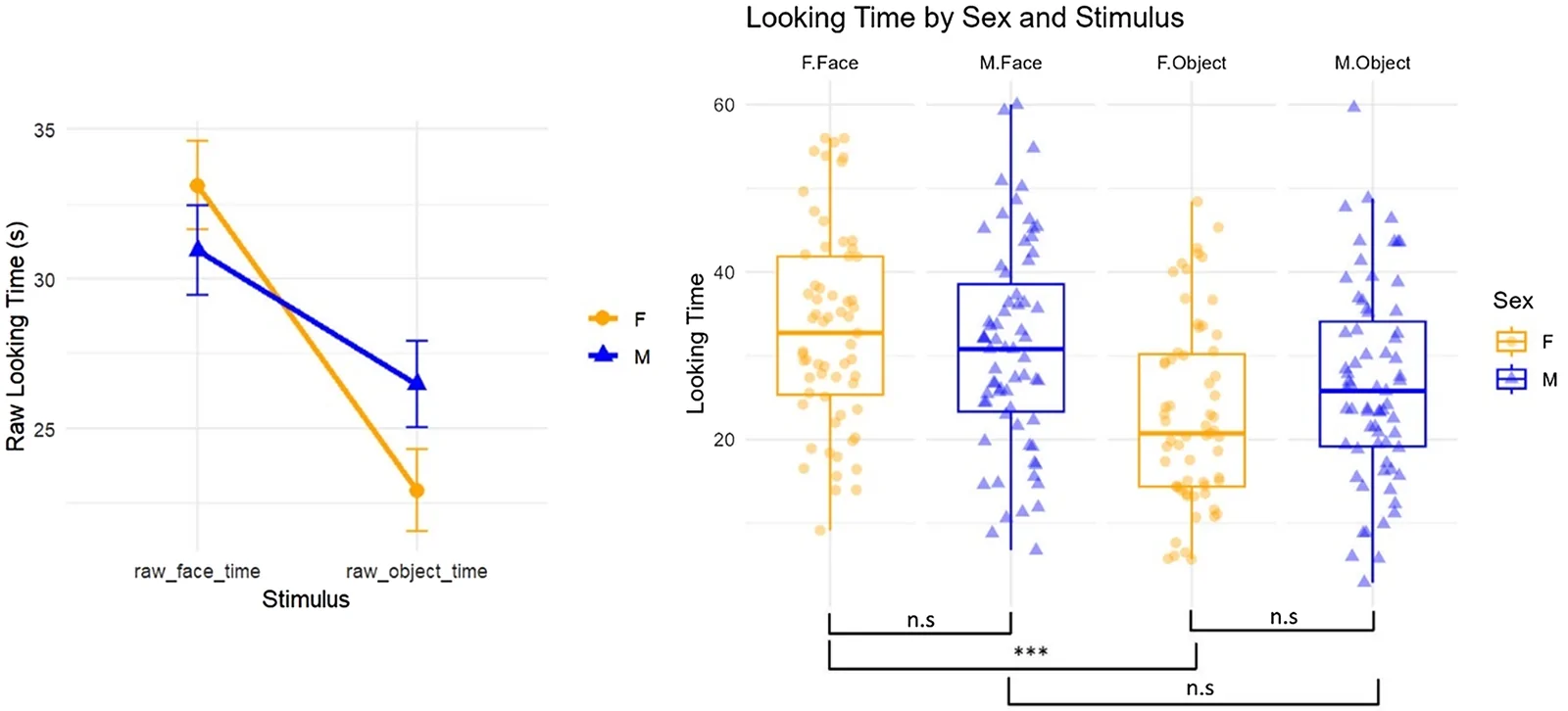
Absolute looking times by sex and stimulus. Figure 4a shows an interaction plot depicting the mean absolute looking times for each stimulus type, separately for males and females. Error bars represent ±1 standard error of the mean. Figure 4b shows the distribution of absolute looking times by sex and stimulus. The central horizontal lines on the boxplots indicate the median, the boxes represent the interquartile range (IQR; 25th–75th percentile), and the whiskers extend to 1.5 × IQR. The triple asterisks indicate the effect that was identified as statistically significant at padj<0.001 based on post-hoc tests, while the label “n.s.” indicates that the effect was not statistically significant

Although not initially analysed by Connellan et al. [20], we additionally assessed sex differences in absolute looking times towards the face and the object. A two-way repeated measures ANOVA revealed a significant main effect of stimulus in predicting looking times (*F*(1, 128) = 8.926, *p* = .003, η² = 0.07). Irrespective of sex, newborn infants spent significantly more time looking at the face (M = 32.00, SD = 12.01) than at the object (M = 24.75, *SD* = 11.43). There was no significant main effect of sex (*F*(1, 128) = 0.353, *p* = .553, η² = 0.00), indicating that overall looking times did not differ by sex irrespective of stimulus type. Importantly, the sex-by-stimulus interaction was significant in predicting looking times (*F*(1, 128) = 5.39, *p* = .022, η² = 0.024). Post-hoc tests show that this interaction was driven by females looking significantly longer (*p*_adj_ = 0.002, *d* = 0.487) at the face (M = 33.20, *SD* = 11.81) compared to the object (M = 22.92, *SD* = 10.94), while males showed no significant differences (*p*_adj_=0.948, *d* = 0.064) in their looking time towards the face (M = 30.94, *SD* = 12.29) and object (M = 26.47, SD = 11.68). There were no significant between-group differences in males’ and females’ absolute looking times towards the face (*M* diff = 2.26, *p*_adj_=0.103, *d* = 0.405) or object (*M* diff = 3.55, *p*_adj_=0.103, *d* = -0.405), though trends in the hypothesised direction were observed (Fig. 4). These findings remained consistent after controlling for postnatal age at testing, gestational age at birth, delivery mode, birth weight, time since last nap and feed, total trial time, number of trials, and time spent looking away from the screen (Supplementary Tables 4 and 5).

### Sensitivity analysis

Additional sensitivity analyses were conducted to assess the robustness and replicability of the findings. First, participants who were initially excluded due to a strong positioning bias (> 90% of looking time directed towards one side) were re-included in the analyses. Results for percentage looking time remained consistent (*t*(141) = 2.11, *p* = .037). Additionally, the sex-by-stimulus interaction for absolute looking times also remained significant (*F*(1, 142) = 4.46, *p* = .036, η² = 0.03), with post-hoc tests similarly demonstrating that females spent longer looking at the face than the object (*p* = .009). Analyses were then conducted re-including participants that were initially excluded due to coder disagreements. Both the percentage looking time (*t*(137) = 2.42, *p* = .017) and absolute looking time (sex-by-stimulus interaction: (*F*(1, 138) = 5.82, *p* = .017, η² = 0.04), female face vs. object post-hoc test: *p* = .002) results remained consistent.

## Discussion

The present study aimed to assess whether behavioural sex differences in attention towards a dynamic face and non-social mechanical object are evident from birth. We found evidence for on-average sex differences in neonatal attention when analysing the *distribution* of attention between the face and object, with females allocating a greater percentage of time looking at a face relative to males. Additionally, when analysing absolute looking times, females showed a preference for the face compared to the non-social object, while males showed no preference for either. No sex differences were identified in absolute looking times towards the face or object, indicating that sex differences are most prominent in the *relative* allocation of attention.

A broader body of previous literature has demonstrated that newborns show a robust preference for faces over non-faces [38–40]. The present findings suggest that this face preference may be more prominent in females. Males, on the other hand, may show a comparatively weaker face preference and distribute their attention more evenly between social and non-social stimuli. These findings are similar to those of Connellan et al. [20], who previously reported that, on average, females spent a significantly proportion of time looking at a face than a non-social object, while males spent a significantly proportion of time looking at a non-social object than a face. The difference in findings for males’ looking times may have emerged due to the different measures (i.e., proportional vs. absolute looking times), since the Connellan et al. [20] study analysed only proportional looking times.

Importantly, the Connellan et al. [20] primarily found *within-*group rather than *between*-group differences. Specifically, that study found that females on average spent a greater proportion of time looking at the face than they did looking at the object, while males spent a greater proportion of time looking at the object than they did looking at the face. They did not, however, identify differences *between* males and females in their looking times toward either stimulus. Critics have highlighted that within-group differences, in the absence of between-group differences, make a less compelling case for sex differences in attention [22, 41]. In the present study, we did not observe between-group sex differences in absolute looking times. However, we identified between-group differences in the percentage of time dedicated towards the social and non-social stimulus. On average, females dedicated a significantly greater percentage of time towards the face compared to males, while males dedicated a significantly greater percentage of time towards the object compared to females. These findings suggest that whilst there are no sex differences in the *absolute* amount of time dedicated towards social and non-social stimuli, on-average sex differences are present in the *distribution* of attention between them.

Given the minimal postnatal experience of newborn infants, it is likely that the observed attentional differences in part reflect the influence of prenatal biological factors. One possible mechanism is endocrine, since during the first and second trimesters of gestation, male fetuses are exposed to approximately 2.5 times more testosterone than female fetuses [42]. This prenatal testosterone surge contributes to the emergence of on-average sex differences not only in physical development, but also in brain structure and later behaviour [9, 13, 43]. Indeed, elevated prenatal testosterone levels are inversely associated with later outcomes such as eye contact, identification of emotional expressions, quality of social relationships, communication, and empathising [14–16, 44]. It is therefore possible that prenatal sex steroid hormones may also contribute to the attentional preferences that we have observed in the present study at birth. However, it is important to note that studies relating variability in prenatal testosterone to subsequent behavioural outcomes have produced inconsistent results and await independent replication [45]. This inconsistency may be because such studies typically measure prenatal testosterone via maternal blood or amniotic fluid in small sample sizes and are therefore not sufficiently powered to consistently detect such associations.

Although likely minimal, it is important to consider the potential influence of early social factors even during the first 3 days of life. Gender socialisation effects may begin prenatally, as research has shown that parents develop sex role consistent expectations of their babies’ attributes based on fetal sex [46]. Postnatally, parents may show subtle differences in their behaviour towards newborn males and females (e.g., in patterns of gaze, speech, or tactile interaction) in ways that may promote a heightened face preference in females. While it is unlikely that early postnatal experiences alone substantially account for these findings, it is possible that they may interact with prenatal biological factors to collectively shape sex differences.

### Alternative mechanisms

There may also be alternative explanations for the sex differences observed in this study. One possibility is that they reflect differences in cognitive maturation. Females, on average, show faster cognitive development than males in childhood [47], and subtle maturational differences have also been reported in the neonatal period. For instance, neonatal male vulnerability – the phenomenon that newborn boys show a higher prevalence of various medical and neurobehavioural complications – is interpretated as evidence of a maturational lag in male neonates [48]. Such maturational differences may contribute to sex differences in early social perception [22, 49] if, for example, females’ faster global maturation enables the more efficient processing of complex visual stimuli such as faces. Relatedly, females were more likely than males to only require one trial to complete the present experiment, which may also indirectly reflect higher global maturation. However, the observed sex differences persisted even after controlling for the number of trials conducted, indicating that this factor alone is unlikely to fully account for the observed findings.

It is also possible that the observed sex differences in attention were influenced by perceptual characteristics (e.g., configuration, colour, contrast, or motion differences between the face and non-social stimuli) rather than by their social versus non-social content. For instance, it is possible that females’ increased attention to faces may not necessarily indicate a face-specific preference. Instead, it could reflect a broader sensitivity to perceptual or social properties that often co-occur with faces, such as top-heavy configurations [50], animate motion [51], or positively valanced expressions. Note, however, that animate motion is not incompatible with this being a social effect, since social stimuli are largely defined by the characteristic of animate motion. Relatedly, given that there is some evidence that female fetuses habituate more quickly than males, it is possible that females’ reduced looking times towards the non-social stimuli may simply reflect faster habituation due to its repetitive, predictable motion [52].

In designing this study, we intentionally did not match these features to preserve ecological validity and capture the inherent differences between social and non-social stimuli (e.g., animate vs. systematic motion), while also avoiding stimuli that might inadvertently resemble a face and activate newborns’ face-processing mechanisms. It is also worth noting that Connellan et al. [20] controlled for several low-level perceptual properties (e.g., size, colour, and shape) in their stimuli and reported comparable findings, suggesting that low-level perceptual differences alone may not fully account for the observed effects. It is nonetheless important to acknowledge that these differences may contribute to the observed sex differences and cannot be fully disentangled from their social/non-social content.

Overall, these findings suggest that, first, sex differences are dependent on the specific stimulus pairings under question as they are not consistently observed across all face versus non-face comparisons [22]. Evidence from the present study and Connellan et al. [20] indicates that sex differences might emerge more clearly when contrasting social stimuli that show the features of natural, animate motion vs. non-social stimuli that show the features of systematic, predictable inanimate motion. Second, sex differences are also dependent on the statistical analytical approach used and how the attentional measure is operationalised (e.g., absolute versus percentage looking times). Absolute looking times measure the absolute duration of attention to a stimulus and do not account for individual difference in the total time an infant spends attending to the screen. In contrast, percentage looking times quantify the relative distribution of attention between competing stimuli while taking into account individual differences in overall attention, which may make them more sensitive to capturing differences in preference. Third, the findings from this study do not rule out the possibility that neonatal sex differences are driven by maturational differences between males and females or perceptual differences between the stimuli, rather than the social vs. non-social content of the stimuli. However, irrespective of whether other factors may underlie this difference, the key conclusion remains that sex differences are present in relative looking preferences very soon after birth. We nonetheless underline the need for careful and nuanced interpretation when considering sex differences in neonatal attention.

### Strengths and limitations

There are several limitations that need to be acknowledged when interpreting the findings of this study. First, as is common in neonatal research, the present study had a high rate of C-sections (~ 47%). This is likely due to the extended hospital stays associated with C-section deliveries, increasing the opportunity for mothers to opt into research. While some studies report no significant postnatal behavioural differences in infants born via C-Sects.  [17, 53, 54], others suggest that it may affect early development by disrupting mother-infant bonding, particularly when performed under general anaesthesia [55]. In recent years, the percentage of C-sections performed under general anaesthesia is relatively low (~ 8%). More important, in our sample, C-section rates did not differ by sex, and the observed results remained consistent after controlling for delivery mode. Therefore, mode of delivery alone cannot account for the observed sex differences.

Second, videos were manually coded by trained observers due to the current methodological challenges associated with applying gaze-tracking in newborns (e.g., issues with calibration and eye detection). Although this approach may introduce some subjectivity, we employed a systematic coding protocol (e.g., by using multiple coders who remained blind to the infant’s sex and to whether each stimulus was presented to the left or right) and achieved good inter-coder reliability. Nevertheless, as more reliable neonatal gaze-tracking technologies emerge, it will be important for future studies to replicating these findings using more objective measures. This would also enable the incorporation of more sophisticated gaze metrics (e.g., time to first look, average fixation duration) and analyses (e.g., time-series analyses). Third, only one experimental trial was conducted due to the limited sustained attention in newborns. Ideally, repeated trials would allow for the assessment of within-subject consistency and would increase statistical power, but this would be challenging to implement in a neonatal sample. Fourth, to prioritise safety, parents were not blindfolded while holding their infants, which may have introduced inadvertent parental biases in infant positioning. It would have been unethical to risk any harm to the baby given their fragility, and unethical to ask parents to abandon their duty to visually monitor their baby’s wellbeing and physical safety. Within these constraints, the researchers carefully monitored each session to ensure the infant was positioned centrally in front of the screen to avoid any biases towards one side. Finally, it is also important to acknowledge that data from both Connellan et al. [20] and the present study were collected in Cambridge, UK. As such, caution should be exercised in generalising these findings to different geographic, cultural, or socioeconomic contexts.

Against these limitations, a strength of the present study is its relatively large sample size. While the sample size is not exceptionally large by today’s psychological research standards, neonatal studies are well known to have small sample sizes (often in single or double digits) due to practical constraints. Moreover, the creation of digital stimuli will facilitate future replication studies. Finally, a key strength is the young age of the infants (mean age = 33 h) which minimises the influence of postnatal experience on the observed findings.

### Perspectives and significance

Ultimately, understanding the origins and developmental timeline of sex differences in the brain and behaviour carries significant implications for both basic science and clinical practice. It is well-evidenced that sex differences are present in the prevalence and presentation of various psychiatric and neurodevelopmental conditions. These differences are hypothesised to arise, at least in part, due to sex differences in neurobiological development. Autism, for instance, is more prevalent in males and shows sex differences in its presentation. One influential theory suggests that this is because autism is characterised by an extreme of cognitive profiles that are, on average, more common in males [56]. A neurobiological account suggests that differences in social cognition in autism may stem from delayed or altered activation of subcortical mechanisms that promote social attention in early infancy [57]. In support of this hypothesis, newborn infants with a high familial likelihood of autism show reduced visual preferences to social stimuli compared to those with a low familial likelihood [58]. One hypothesis that may be investigated further is whether sex differences in early social attention may be further amplified in autism, partly contributing to the observed sex differences in its prevalence and presentation.

## Conclusions

In summary, the present findings demonstrate that sex differences in the relative allocation of attention towards dynamic faces and mechanical objects are evident within the first days of life. These differences appear to be dependent on the specific stimulus pairings and the analytical approach used. By virtue of their limited postnatal experience, it is possible that these sex differences may emerge partly due to prenatal biological factors, though alternative maturational or perceptual explanations remain plausible. Ultimately, understanding the origins and developmental timeline of sex differences in the brain and behaviour has important implications for both basic science and clinical practice. Validating the relevance of the current findings to clinical research will be an important future step.

## Supplementary Information

Below is the link to the electronic supplementary material.

Supplementary Material 1

## Data Availability

No datasets were generated or analysed during the current study.
