# Supplementary material for: Sex differences in face looking preferences at birth
Source: Biol Sex Differ. 2026 Sep 1;17:155. doi: 10.1186/s13293-026-00976-9 (PMC13591834; doi:10.1186/s13293-026-00976-9)
Supplement: Supplementary file 1 [file 13293_2026_976_MOESM1_ESM.zip › Supplementary materials/Supplementary Tables sex diffs at birth dev sci.docx]

**Supplementary Materials: Sex Differences in Social Attention at Birth**

**Supplementary Table 1**

|  | **t statistic** | **df** | ***p*** | **Cohen’s *d*** |
| --- | --- | --- | --- | --- |
| Postnatal age (hours) | 0.80 | 134 | 0.46 | 0.12 |
| Gestational age at birth (weeks) | -0.91 | 134 | 0.36 | -0.14 |
| Birth weight (grams) | -2.55 | 134 | 0.01 | -0.45 |
| Maternal age (years) | -0.86 | 134 | 0.39 | -0.12 |
| Paternal age (years) | -0.64 | 130 | 0.52 | 0.06 |
| Trial time | -0.53 | 128 | 0.60 | -0.09 |
| Time spent looking away from the screen (seconds) | 0.39 | 128 | 0.70 | 0.07 |
|  | **Pearson’s chi square** | **df** | ***p*** |  |
| Delivery mode | 4.07 | 5 | 0.54 | - |
| Left-right positioning of stimulus | 0.00 | 1 | 0.97 |  |
| Number of trials | 7.52 | 2 | 0.02 | - |

Sex differences in sample characteristics

*Note.* Supplementary Figure 1 reports sex differences in sample characteristics assessed via t-tests for continuous variables and chi-square tests for categorical variables. A positive t-statistic indicates greater values in females, while a negative t-statistic indicates greater values in males.

**Supplementary Table 2**

*Predictors of percentage looking time towards the face*

| **Predictor** | **Estimate** | **Std. Error** | ***p*** |
| --- | --- | --- | --- |
| GA at birth | -1.18 | 1.67 | 0.48 |
| Trial time | -0.25 | 0.51 | 0.63 |
| Time looking away | -0.04 | 0.18 | 0.82 |
| Delivery mode: C-section | -25.86 | 19.08 | 0.18 |
| Delivery mode: Vaginal, breech | -10.92 | 18.94 | 0.57 |
| Delivery mode: Vaginal, forceps | -2.66 | 5.47 | 0.63 |
| Delivery mode: Vaginal, spontaneous | 3.56 | 3.75 | 0.34 |
| Delivery mode: Vaginal, vacuum | -5.52 | 9.69 | 0.57 |
| Birth weight | -0.01 | 0.00 | 0.19 |
| Time since last feed | 0.00 | 0.02 | 0.99 |
|  |  |  |  |
| Time since last nap | 0.01 | 0.04 | 0.74 |
| Postnatal age | 0.07 | 0.11 | 0.54 |
| Number of trials | -3.66 | 3.03 | 0.23 |

*Note.* Results from the multiple linear regression predicting percentage looking time towards the face. The table displays unstandardised regression coefficients (Estimates), standard errors, and associated p-values. Positive estimates indicate that the predictor is linked with higher looking times towards the face.

**Supplementary Table 3**

*ANCOVA on sex differences in percentage looking time towards the face*

|  | **df numerator** | **df denominator** | ***F* statistic** | ***p*** |
| --- | --- | --- | --- | --- |
| **Sex** | **1** | **115** | **5.20** | **0.02** |
| Gestational age at birth | 1 | 115 | 4.13 | 0.04 |
| Seconds looking away | 1 | 115 | 0.39 | 0.54 |
| Trial time | 1 | 115 | 0.01 | 0.92 |
| Delivery mode | 5 | 115 | 0.62 | 0.69 |
| Birth weight | 1 | 115 | 1.53 | 0.22 |
| Time since last feed | 1 | 115 | 0.03 | 0.87 |
| Time since last nap | 1 | 115 | 0.31 | 0.58 |
| Postnatal age | 1 | 115 | 0.12 | 0.73 |
| Number of trials | 1 | 115 | 0.69 | 0.41 |

*Note.* Results of ANCOVA examining the effect of sex on percentage looking time towards the face, controlling for gestational age at birth, trial time, time spent looking away from the screen, delivery mode, birth weight, time since last feed and nap, postnatal age, and number of trials. Reported are degrees of freedom (df), F values, and associated *p* values. Sex differences remain significant after controlling for all covariates.

**Supplementary Table 4**

*ANCOVA on sex x stimulus interaction on raw looking times*

|  | **df numerator** | **df denominator** | ***F* statistic** | ***p*** |
| --- | --- | --- | --- | --- |
| Sex | 1 | 243 | 0.79 | 0.37 |
| stimulus | 1 | 243 | 28.04 | <.001 |
| **Sex × stimulus** | **1** | **243** | **4.39** | **0.04** |
| Gestational age at birth | 1 | 243 | 0.26 | 0.61 |
| Delivery mode | 5 | 243 | 0.16 | 0.98 |
| Birth weight | 1 | 243 | 0.19 | 0.66 |
| Time since last feed | 1 | 243 | 0.00 | 0.95 |
| Time since last nap | 1 | 243 | 0.12 | 0.73 |
| Postnatal age | 1 | 243 | 0.23 | 0.64 |
| Number of trials | 1 | 243 | 2.07 | 0.15 |
| Seconds looking away | 1 | 243 | 37.82 | <.001 |
| Trial time | 1 | 243 | 1.35 | 0.25 |

*Note.* Results of ANCOVA examining the effect of sex, stimulus, and sex x stimulus interactions on raw face looking time, controlling for gestational age at birth, trial time, time spent looking away from the screen, delivery mode, birth weight, time since last feed and nap, postnatal age, and number of trials. Reported are degrees of freedom (df), F values, and associated *p* values. Sex*stimulus interaction remains significant after controlling for all covariates.

**Supplementary Table 5**

*Post hoc t-tests on ANCOVA examining sex x stimulus interaction on raw looking times*

| **Contrast** | **Estimate** | **SE** | **df** | **t ratio** | ***p*_adj_** |
| --- | --- | --- | --- | --- | --- |
| F face time - M face time | 2.61 | 2.03 | 243 | 1.28 | 1.00 |
| F face time - F object time | 10.21 | 1.97 | 243 | 5.19 | <.001 |
| F face time - M object time | 7.08 | 2.03 | 243 | 3.48 | 0.004 |
| M face time - F object time | 7.60 | 2.03 | 243 | 3.74 | 0.001 |
| M face time - M object time | 4.47 | 1.91 | 243 | 2.34 | 0.12 |
| F object time - M object time | -3.13 | 2.03 | 243 | -1.54 | 0.75 |

*Note.* Post-hoc pairwise comparisons of adjusted mean looking times based on the ANCOVA in Supplementary Figure 4 on raw looking times. Reported are estimates (representing the difference in raw looking time between each pair), *p* values (Bonferroni adjusted), degrees of freedom (df), and t ratios. Contrasts were computed between all Sex × Stimulus combinations and Bonferroni adjustments were made to correct for multiple comparisons.
